# Skeletal-Muscle-Specific Overexpression of Chrono Leads to Disruption of Glucose Metabolism and Exercise Capacity

**DOI:** 10.3390/life12081233

**Published:** 2022-08-15

**Authors:** Shiyi He, Lu Yan, Rongxin Zhu, Hao Wei, Jianxiong Wang, Lan Zheng, Ying Zhang

**Affiliations:** 1 Key Laboratory of Physical Fitness and Exercise Rehabilitation of Hunan Province, College of Physical Education, Hunan Normal University, Changsha 410012, China; 2 School of Sport Science, Beijing Sport University, Beijing 100084, China; 3 Shanghai Research Institute of Sports Science, Shanghai 200030, China; 4 Faculty of Health, Engineering, and Sciences, University of Southern Queensland, Toowoomba, QLD 4350, Australia

**Keywords:** glucose metabolism, skeletal muscle, Chrono, exercise capacity, mice

## Abstract

Disruption of circadian rhythms is related to disorders of glucose metabolism, and the molecular clock also exists in skeletal muscle. The ChIP-derived repressor of network oscillator (Chrono) and brain and muscle ARNT-like 1 (Bmal1) are core circadian components. Chrono is considered to be the repressor of Bmal1, and the Chrono–Bmal1 pathway is important in regulating the circadian rhythm; it has been speculated that this pathway could be a new mechanism for regulating glucose metabolism. The purpose of this study was to investigate the effects of Chrono on glucose metabolism in skeletal muscle and exercise capacity by using mice with skeletal-muscle-specific overexpression of Chrono (Chrono TG) and wild-type (WT) mice as the animal models. The results of this cross-sectional study indicated that the Chrono TG mice had an impaired glucose tolerance, lower exercise capacity, and higher levels of nonfasted blood glucose and glycogen content in skeletal muscle compared to WT mice. In addition, the Chrono TG mice also showed a significant increase in the amount of Chrono bound to Bmal1 according to a co-IP analysis; a remarkable decrease in mRNA expression of *Tbc1d1*, *Glut4*, *Hk2*, *Pfkm*, *Pdp1*, *Gbe1*, and *Phka1*, as well as in activity of Hk and protein expression of Ldhb; but higher mRNA expression of *Pdk4* and protein expression of Ldha compared with those of WT mice. These data suggested the skeletal-muscle-specific overexpression of Chrono led to a greater amount of Chrono bound to Bmal1, which then could affect the glucose transporter, glucose oxidation, and glycogen utilization in skeletal muscle, as well as exercise capacity.

## 1. Introduction

Skeletal muscle comprises approximately 40% of the total body weight in humans [1] and is responsible for over 80% of glucose uptake [2]. The uptake and disposal of glucose by skeletal muscle are essential for the body to maintain blood glucose homeostasis. Dysfunction of glucose metabolism resulting in insulin resistance in skeletal muscle is the primary defect of type 2 diabetes. Recent studies have shown that disruption of the circadian rhythm has also led to dysfunctional glucose metabolism of skeletal muscle [3,4], in addition to unhealthy diets [5,6,7] and physical inactivity [8], which are also related to glucose disorders in skeletal muscle.

Generated endogenously in an organism, the circadian rhythm is responsible for orchestrating many daily metabolic activities, including the 24-hour period of glucose metabolism. According to the location of oscillators in mammals, circadian rhythms are divided into central and peripheral clocks. The central clock lies in the suprachiasmatic nucleus of the hypothalamus, and the peripheral clocks are in the peripheral organs, including the liver, heart, skeletal muscle, and possibly other organs [9]. At the molecular level, circadian rhythms are driven by the molecular clock, a transcriptional–translational feedback loop (TTFL) [10], which involves the core clock molecules, such as Bmal1, circadian locomotor output cycles kaput (Clock), and others. Clock and Bmal1 heterodimerize in the nucleus, which can activate the transcription of genes related to biological processes (such as glucose metabolism and autophagy) by binding E-box elements (CACGTG) in their regulatory regions [11]. At the same time, the molecular clock also exists in skeletal muscle. Recent research has demonstrated that circadian rhythms in skeletal muscle are necessary to regulate glucose metabolism. Using high-resolution time-course analysis, 1628 circadian genes involved in carbohydrate metabolism were identified in skeletal muscle [12]. The core clock molecular Bmal1 regulated muscle insulin sensitivity [13], and muscle-specific loss of Bmal1 decreased glucose metabolism and affected systemic glucose homeostasis [14].

*Chrono*, also known as *GM129*, circadian associated repressor of transcription (*Ciart*), or *C1orf51*, is a conserved gene in mammals and is widely distributed in the skeletal muscle, liver, hypothalamus, and other organs. Goriki et al. found that *Chrono* mRNA expression oscillated during a biorhythmic cycle of approximately 24 h and was opposite to the oscillatory expression of *Bmal1* mRNA in mouse skeletal muscle [15]. Chrono knockout mice had longer periods of activity rhythms, waking up progressively later each day [16]. More evidence has also shown that Chrono repressed Bmal1 transcriptional activity to interact with the C-terminus of the Bmal1 protein [17]. Thus, Chrono is considered to be the repressor of Bmal1, and the Chrono–Bmal1 pathway is important in regulating the circadian rhythm. Moreover, it has also been speculated that the Chrono–Bmal1 pathway may be a new mechanism for regulating glucose metabolism. However, few studies of the functions of core molecular clock genes in skeletal muscle, especially for *Chrono*, have been documented. Therefore, the purpose of this study was to investigate the effects and mechanisms of Chrono on glucose metabolism and exercise capacity by using a mouse model of skeletal-muscle-specific Chrono overexpression.

## 2. Method

### 2.1. Animals

Chrono muscle-specific overexpression mice (10 weeks old; 6 males and 2 females) of C57BL/6N background were purchased from Cyagen Biosciences Inc. (Santa Clara, CA, USA). The hybridized mice of Myf5-Cre and Chrono flox/flox created using CRISPR/Cas-mediated genome engineering were used to investigate the role of Chrono in skeletal muscle directly. PCR analysis was performed by the company, and the results are presented in the Supplementary Materials. The Chrono muscle-specific overexpression mice and their littermate controls are herein referred to as Chrono TG and WT mice, respectively. All mice were housed in a temperature- and light-controlled environment (20–25 °C and 12 h light–dark cycle) with food and water ad libitum. When the age of mice was 12 weeks, they were chosen to conduct the endurance exercise capacity test. After one week of measurement, the glucose tolerance test was conducted. At 15 weeks, all mice were sacrificed by cervical dislocation between 7:00 p.m. and 10:00 p.m., and all other measurements were carried out.

### 2.2. Endurance Exercise Capacity Test

All mice were familiarized with treadmill running for three days. After that, an endurance exercise capacity test was conducted between 7:00 p.m. and 10:00 p.m. using an incremental treadmill with a Comprehensive Lab Animal Monitoring System (Columbus Instruments International Corp., Columbus, OH, USA). The test began with an incline of 5° and a speed of 10 m/min for 5 min. After this initial phase, the speed was progressively increased by 3 m/min every 3 min until the mouse could not remain at speed and lasted over 10 s on the electric grid at the rear of the equipment [18]. Once exhaustion was reached, the power of the shock grid was turned off, and running duration, distance, and maximal oxygen uptake (VO_2max_) were recorded.

### 2.3. Blood Lactate

Tail venous blood was taken before and after the endurance exercise capacity test and put into a blood lactate analyzer (Biosen C-Line, EKF diagnostic GmbH, Barleben, Germany) for measurement.

### 2.4. Glucose Tolerance Test (GTT)

After the endurance exercise capacity test, the mice rested for a week and had free access to food and water. The GTT was conducted after 8 h of fasting. All mice received an intraperitoneal injection of D-glucose (1 g/kg of body weight) at 8:00 p.m. Blood glucose levels were determined with a blood glucose meter (GA-6, Sannuo, Changsha, China) from a tail nick taken at 0 (before injection), 15, 30, 45, 60, 90, and 120 min after glucose injection. The area under the curve (AUC) of the GTT was calculated.

### 2.5. Nonfasted Blood Glucose and Insulin

Before the mice were euthanized, nonfasted blood was collected from their orbits. Blood glucose was measured using the same blood glucose meter mentioned above. A commercial plasma insulin Elisa kit (GEL2579-A, Genelab Company, China) was used to measure the nonfasted plasma insulin concentration.

### 2.6. Muscle Glycogen Content and Activity of Glycogen Synthase (Gys) and Hexokinase (Hk)

Gastrocnemius (GAS) muscle was used to measure glycogen content using a glycogen content detection kit (BC0345, Solarbio, Beijing, China), while the activity of Gys and Hk were determined via a Gys assay kit (BC3335, Solarbio, Beijing, China) and an Hk assay kit (BC0745, Solarbio, Beijing, China), respectively.

### 2.7. Real-Time Quantitative PCR (RT-qPCR) Analysis

RNA was isolated from GAS samples from the WT and Chrono TG mice. Briefly, 50 mg of GAS tissue was homogenized in 1 mL TRIzol reagent (Life Technologies, Eugene, OR, USA). RNA was carried out following the manufacturer’s instructions. About 1 μg of total RNA was used to synthesize cDNA using a kit (FSQ-101; Toyobo Co., Ltd., Osaka, Japan). The RT-qPCR was performed in an ABI 7500 Real-Time PCR System (Thermo Scientific, Inc., Waltham, MA, USA) using the SYBR Green Real-Time PCR Master Mix kit (Toyobo Co., Ltd., Osaka, Japan). The commercial primers from Qiagen (Hilden, Germany) of *Chrono* (QT01533749), glucose transporter type 4 (*Glut4*, QT01044946), glycogen branching enzyme 1 (*Gbe1*, QT00252924), phosphorylase kinase regulatory subunit alpha 1 (*Phka1*, QT00143514), hexokinase 2 (*Hk2*, QT00155582), muscle phosphofructokinase (*Pfkm*, QT00159754), pyruvate dehydrogenase phosphatase catalytic subunit 1 (*Pdp1*, QT01165374), TBC1 domain family member 1 (*Tbc1d1*, QT00156898), pyruvate dehydrogenase kinase 4 (*Pdk4*, QT00157248), and 18S ribosomal RNA (Rn18s, QT02448075) were used. The mRNA primers of glycogen synthase 1 (*Gys1*) were synthesized by Thermo Fisher, and the primer sequences are shown in Table 1. The expression of each sample was calculated using the 2^−ΔΔCt^ method, as described previously [19].

### 2.8. Western Blot

Total proteins were isolated from 50 mg of GAS using RIPA protein extraction reagents (P0013B; Beyotime, Inc., Shanghai, China). Proteins were separated on Bolt 4–12% Bis-Tris plusGels (NW04125BOX; Thermo Fisher Scientific, Inc., Waltham, MA, USA) and subsequently transferred to a nitrocellulose membrane using iBlot gel transfer stacks (IB23001; Thermo Fisher Scientific, Inc., Waltham, MA, USA). Immunoblots were probed using the following antibodies: Chrono (1:500, PA5-55643, Thermo Fisher Scientific, Inc., Waltham, MA, USA), Ldha (1:2000, 66287-1-Ig; Proteintech Group, Chicago, IL, USA), Ldhb (1:10,000, 66425-1-Ig; Proteintech Group, Chicago, IL, USA), and β-actin (1:1000, SC-47778, Santa Cruz Biotechnology, Dallas, TX, USA). The density of protein bands was analyzed using Bio-Rad imaging software (Bio-Rad Laboratories, Hercules, CA, USA).

### 2.9. Co-Immunoprecipitation (Co-IP) Assay

Total protein was extracted from GAS. The lysate was precleared with protein A/G agarose at 4 °C for 2 h. Then, 3 ug of the primary antibody anti-Bmal1 (SC-365645, Santa Cruz Biotechnology, Dallas, TX, USA) was added into lysate containing 1 mg total protein and rotated at 4 °C overnight. The following day, we added the protein A/G agarose and rotated for 4 h. The samples were washed with cold TNE buffer for four times and collected for Western blotting.

### 2.10. Data Processing and Statistical Methods

All data were processed using SPSS 21.0 statistical software, and the results were analyzed using an independent samples *t*-test. The results were expressed as mean ± standard error (X ± SE); *p* < 0.05 and *p* < 0.01 indicate statistically significant and highly significant, respectively.

## 3. Results

### 3.1. Food Intake, Body Weight, and Adipose Tissue Weight

**Figure 1:**
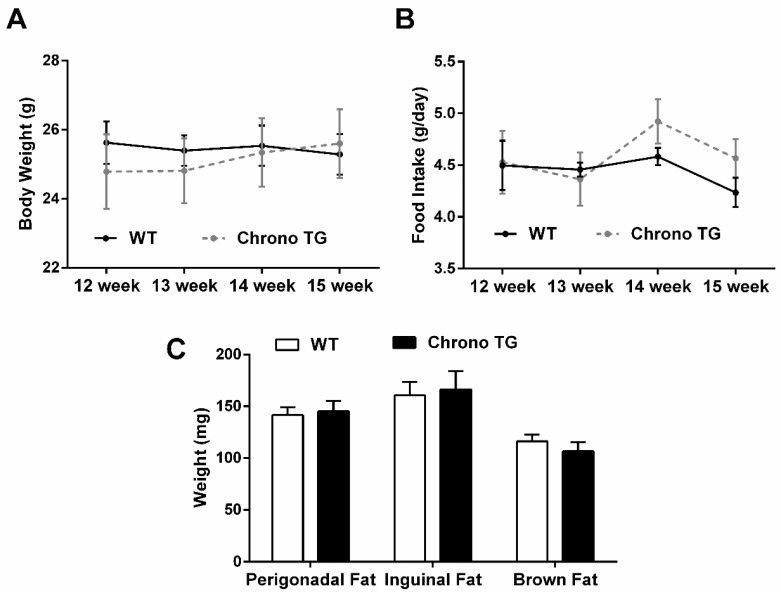
Effects of Chrono overexpression of skeletal muscle on body weight (**A**), food intake (**B**), and adipose tissue weight (**C**).

There was no significant difference in food intake and body weight of Chrono TG mice compared with WT mice (Figure 1A,B). The weight of perigonadal, inguinal, and brown fat was also not different between the two groups (Figure 1C).

### 3.2. GTT, Nonfasted Blood Glucose, and Insulin

**Figure 2:**
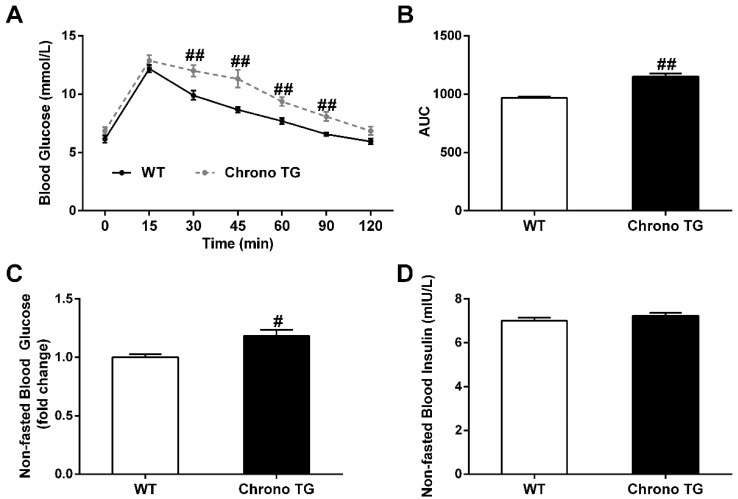
Effects of Chrono overexpression of skeletal muscle on systemic glucose homeostasis. Glucose tolerance is depicted as blood glucose versus time post-glucose injection (**A**) and AUC (**B**). Nonfasted blood glucose (**C**). Nonfasted blood insulin (**D**). # *p* < 0.05, ## *p* < 0.01 vs. WT.

Chrono TG mice had higher blood glucose levels after an intraperitoneal glucose injection compared to those of WT mice (Figure 2A). Furthermore, the AUC of Chrono TG mice was greater than that of WT mice (Figure 2B). There was no significant difference in nonfasted blood insulin between the two groups (Figure 2D), but the nonfasted blood glucose of Chrono TG mice exhibited a significant increase (Figure 2C).

### 3.3. Exercise Capacity and Blood Lactate Content

**Figure 3:**
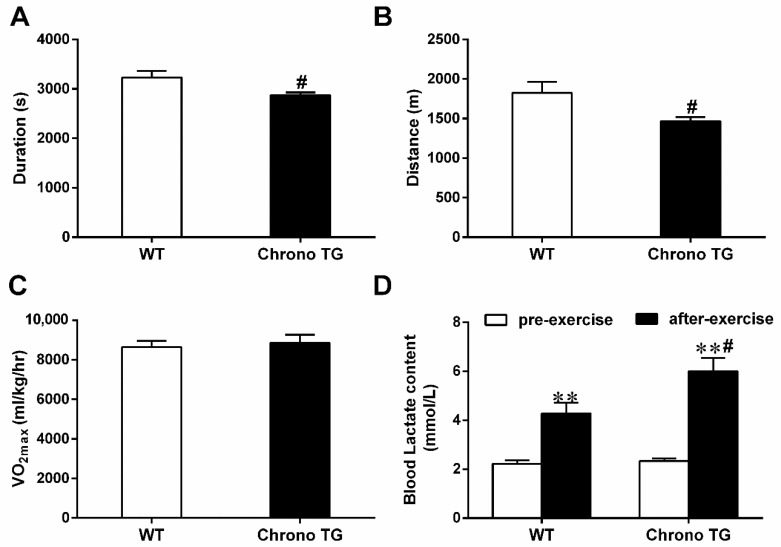
Effects of Chrono overexpression of skeletal muscle on the running duration (**A**), running distance (**B**), and VO_2max_ (**C**), and blood lactate (**D**). # *p* < 0.05, Chrono TG vs. WT. ** *p* < 0.01, after-exercise vs. pre-exercise.

In the Chrono TG and WT mice, the running duration was 2875 ± 55 and 3231 ± 130 s (Figure 3A), respectively; the running distance was 1468 ± 49 m and 1825 ± 140 m (Figure 3B), respectively. The above results indicated a significant decrease in the endurance exercise performance of the Chrono TG mice, though there was no difference in VO_2max_ between the two groups (Figure 3C). For the exercise-induced blood lactate level, the Chrono TG mice had a higher blood lactate level than that of WT mice (Figure 3D).

### 3.4. The mRNA Expression of Chrono, Tbc1d1, Glut4, Hk2, Pfkm, Pdp1, and Pdk4; and the Hk Activity and the Amount of Chrono Bound to Bmal1

**Figure 4:**
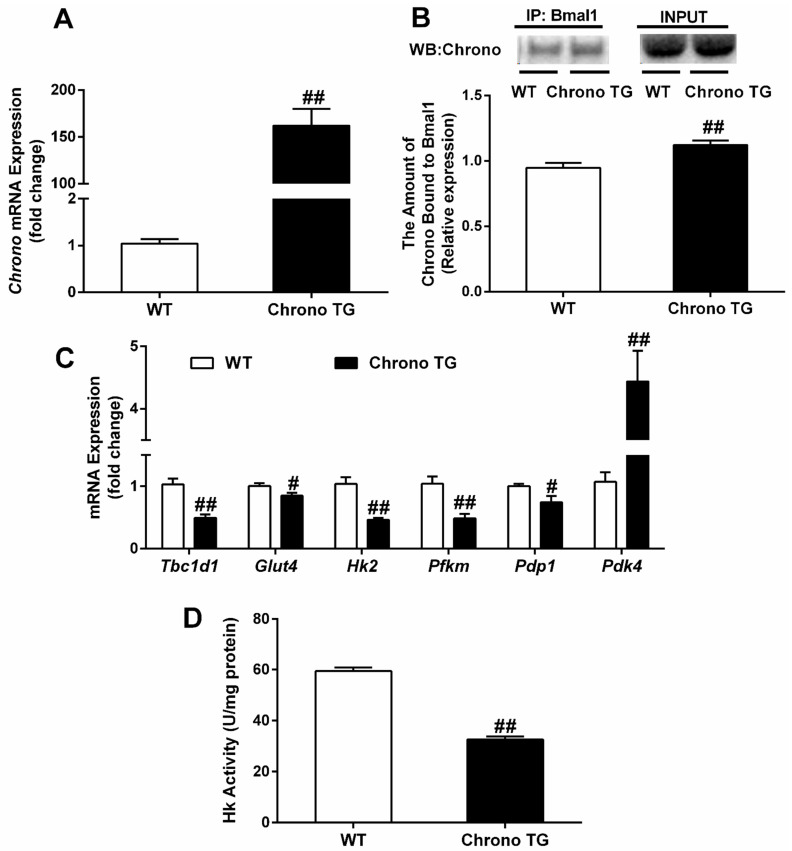
Effects of Chrono overexpression of skeletal muscle on the amount of Chrono bound to Bmal1 (**B**), the mRNA expression of *Chrono* (**A**), *Tbc1d1*, *Glut4*, *Hk2*, *Pfkm*, *Pdp1* and *Pdk4* (**C**) and Hk activity (**D**) in skeletal muscle. # *p* < 0.05, ## *p* < 0.01, Chrono TG vs. WT.

The RT-qPCR confirmed the efficiency of *Chrono* overexpression in skeletal muscle (Figure 4A). In addition, the mRNA expression of *Tbc1d1*, *Glut4*, *Hk2*, *Pfkm*, and *Pdp1* and Hk activity were significantly reduced, but the amount of Chrono bound to Bmal1 and *Pdk4* mRNA expression were increased in Chrono TG mice compared with those of WT mice (Figure 4B–D).

### 3.5. The mRNA Expression of Gys1, Gbe1, and Phka1; Activity of Gys; Glycogen Content; as well as Protein Expression of Ldha and Ldhb

**Figure 5:**
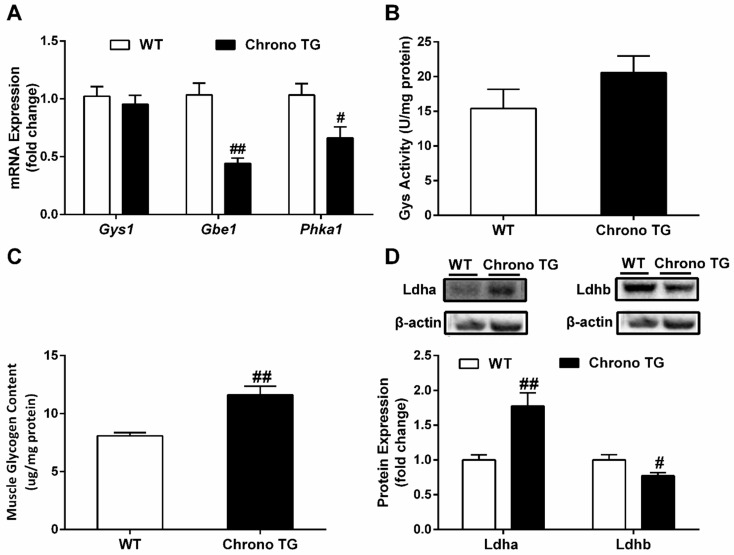
The effect of Chrono overexpression of skeletal muscle on mRNA expression of genes involved in *Gys1*, *Gbe1*, and *Phka* (**A**), Gys activity (**B**), glycogen content (**C**), and protein expression of Ldha and Ldhb (**D**) in skeletal muscles. # *p* < 0.05, ## *p* < 0.01, Chrono TG vs. WT.

The mRNA expressions of *Gbe1* and *Phka1* were significantly reduced in Chrono TG mice compared with those of WT mice (Figure 5A). Meanwhile, there was a significant increase in glycogen content (Figure 5C). Furthermore, mRNA expression of *Gys1* and activity of Gys had no change between the two groups (Figure 5A,B). In addition, a higher protein expression of Ldha and a lower expression of Ldhb protein were observed in the Chrono TG mice compared to WT mice (Figure 5D).

## 4. Discussion

The main finding of the present study revealed that skeletal-muscle-specific overexpression of Chrono increased blood glucose levels in a nonfasted state, but decreased glucose tolerance and aerobic exercise capacity significantly. Moreover, the Chrono TG mice exhibited a greater amount of Chrono bound to Bmal1, a lower mRNA expression of genes related to glucose transport (*Tbc1d1* and *Glut4*), crucial rate-limiting genes of glucose oxidation (*Hk2*, *Pfkm*, *Pdp1*, and *Pdk4*), and glycogen-metabolism-related genes (*Gbe1* and *Phka1*), as well as a higher glycogen level in skeletal muscle, compared to those of WT mice. To our knowledge, this was the first study to show that the skeletal-muscle-specific overexpression of Chrono may affect glucose transport, glucose oxidation, and glycogen utilization in skeletal muscle.

It is well known that Glut4 is the main protein involved in glucose transport in skeletal muscle. It can be translocated to the cell membrane from cytoplasm, and then carries glucose into the cell upon stimulation, such as by exercise or insulin [20]. The phosphorylation of Tbc1d1 protein encoded by *Tbc1d1* gene can promote the translocation of Glut4 [21]. It has been reported that Tbc1d1 knockout rats [22] and skeletal-muscle-specific knockout Glut4 mice [23] had a decreased glucose absorption capability. In addition, Tbc1d1 knockout mice showed lower levels of Glut4 expression, GTT, and insulin-stimulated glucose uptake in skeletal muscle [24]. In our study, the overexpression of skeletal muscle Chrono decreased the expression of the *Tbc1d1* and *Glut4* genes, which might impair skeletal muscles’ ability to transport glucose. Moreover, Hk and Pfk are the rate-limiting enzymes in the glycolytic pathway of skeletal muscle. With two isoforms of Hk1 and Hk2, Hk catalyzes the conversion of glucose to glucose 6-phosphate, and Hk2 is found primarily in insulin-sensitive tissues such as the heart, skeletal muscle, and adipose tissue [25]. Winther et al. demonstrated that the transfection of small interfering RNA targeting Hk2 reduced brown preadipocyte cells’ glucose uptake capacity by diminishing glycolytic flux [26]. Pfkm is the main form of Pfk in skeletal muscle. Deleting the *Pfkm* gene leads to a decrease in systemic glucose tolerance and insulin resistance [27]. Reduced expression of Hk and Pfkm in skeletal muscle provides strong evidence for a significantly lower flux through glycolysis [28]. In addition, the mammalian pyruvate dehydrogenase complex (PDC) is a mitochondrial multienzyme complex that connects glycolysis to the tricarboxylic acid cycle by catalyzing pyruvate oxidation to produce acetyl-coenzyme A (acetyl-CoA), NADH, and CO_2_ [29]. Pyruvate dehydrogenase kinase (Pdk) and pyruvate dehydrogenase phosphatase (Pdp) are considered the main control mechanism of mammalian PDC activity. Pdp dephosphorylates PDC to stimulate its activity, while Pdk inhibits its activity by phosphorylating PDC. Pdp1, an isoform of Pdp, is mainly distributed in skeletal muscle, and promotes the entry of pyruvate into the carboxylic acid cycle for aerobic metabolism [30,31]. Pdk4 is also the primary form of Pdk distributed in skeletal muscle, and an increase in Pdk4 inhibits aerobic glucose metabolism, which in turn allows the conversion of pyruvate to lactate [32]. Mori et al. discovered a link between decreased glucose tolerance and elevated Pdk4 levels in mice treated with angiotensin II [33]. It is interesting that the overexpression of Chrono also resulted in a significant reduction in the mRNA expression of the glucose-oxidation-related genes *Hk2*, *Pfkm*, and *Pdp1* and Hk enzyme activity, but an increased *Pdk4* mRNA expression in the gastrocnemius muscle (Figure 4C,D), implying that Chrono overexpression in skeletal muscle could hinder glucose oxidation and decrease the ability to metabolize glucose.

Glycogen is a branched polymer of glucose that acts as an energy store in times of nutritional sufficiency for utilization when needed. The glycogen branching enzyme (Gbe), which is encoded by the *Gbe1* gene, catalyzes the transfer of alpha-1,4-linked glucosyl units from the outer end of a glycogen chain to an alpha-1,6 position on the same or a neighboring glycogen chain. Branching of the chains is essential to increase the solubility of the glycogen molecule and, consequently, to reduce the osmotic pressure within cells. The highest levels of this enzyme are found in the liver and muscle. Mutations in this gene are associated with glycogen storage disease IV (also known as Andersen’s disease) [34]. Phosphorylase kinase is an essential regulator of glycogen catabolism, and its primary function is to promote glycogen breakdown [35]. Phosphorylase kinase regulatory subunit alpha 1 (Phka1) is a protein-coding gene that encodes the skeletal muscle isoform [36]. Akira et al. found a significant increase in the mRNA expression of *Gbe1* and *Phka1* in both mouse skeletal muscle and C2C12 myotubes after NF-E2-related factor 2 (Nrf2) induction, accompanied by a decreased glycogen content [37]. The downregulation or deletion of the skeletal muscle *Gbe1* and *Phka1* genes could result in impaired enzymatic activity and reduced skeletal muscle glycogen utilization [38,39,40,41]. In addition, glycogen synthase (Gys), a rate-limiting enzyme for glycogen synthesis, is encoded by the *Gys1* gene and catalyzes the addition of glucose monomers to the growing glycogen molecule through the formation of alpha-1,4-glycoside linkages [42]. An elevated Gys activity due to *Gys1* mutation is associated with abnormal muscle glycogen storage in horses [43], and the selective suppression of muscle Gys1 resulted in a considerable reduction in the glycogen content in mouse skeletal muscle [44]. Our study demonstrated that the overexpression of Chrono in skeletal muscle induced a significant increase in the glycogen level and a decrease in mRNA expression of *Gbe1* and *Phka1*, but no significant change in *Gys1* mRNA expression or activity of Gys (Figure 5A–C). These results suggested that the overexpression of Chrono in skeletal muscle might also inhibit glycogen catabolism in skeletal muscle, except for the reduction in glucose oxidation.

We generally surmised that overexpression of Chrono in skeletal muscle might decrease glucose and glycogen metabolism. The data were consistent with our findings of higher non-fasting glucose level and depressed glucose tolerance in Chrono TG mice (Figure 2A–C). Simultaneously, reduced endurance exercise capacity in Chrono TG mice (Figure 3A,B) may also be attributed to the drop in glucose and glycogen utilization in skeletal muscle. The phenomenon is similar to the symptoms of patients/animals with glycogen storage disease, who have an abnormal accumulation of skeletal muscle glycogen and exercise intolerance [45,46]. To obtain the evidence about the effects of Chrono overexpression of skeletal muscle on exercise capacity, we further examined blood lactate level before and after the exercise capacity test and the protein expression of Ldh-a and Ldh-b, which catalyze pyruvate to lactate and lactate to pyruvate conversions respectively [47], in skeletal muscle. In the present study, it is notable that the blood lactate content in the Chrono TG group was higher than that of WT group after the endurance exercise (Figure 3D). Conceivably, the outcome of higher expression of Ldha and the lower expression of Ldhb in Chrono TG mice than those of WT mice illustrated that overexpression of Chrono in skeletal muscle might have great effects on lactate production and increase blood lactate level during exercise (Figure 5D), which could be one of the important reasons resulting in a low endurance exercise capacity.

The role of Chrono in glucose/glycogen metabolism may be connected to the interaction of Chrono with Bmal1, resulting in the suppressed transcriptional activity of Bmal1. As a transcription factor and biorhythm component, Bmal1 binds to the E-box motif on the promoters of its target genes and regulates their transcription. Meanwhile, it has been demonstrated that Bmal1 influences the expression of genes involved in glucose metabolism. Dyar et al. discovered that skeletal muscle Bmal1 deletion dramatically reduced Glut4, Hk2, and Pdp1 protein expression, as well as *Pdp1* mRNA expression [48]. Harfmann et al. also found that skeletal-muscle-specific Bmal1 knockout mice had significantly lower mRNA expression of *Hk2* and *Pfkm* compared to WT mice [14]. Furthermore, according to the Eukaryotic Promoter Database [49], it was speculated that *Glut4*, *Tbc1d11*, *Gbe1*, *Phka1*, *Hk2,* and *Pdp1* were the target genes of Bmal1 (*p* < 0.001). In our research, we found that the amount of Chrono bound to Bmal1 showed a significant increase (Figure 4B), and the mRNA expression of *Glut4*, *Tbc1d1*, *Gbe1*, *Phka1*, *Hk2*, and *Pdp1* showed a remarkable reduction in Chrono TG mice compared with WT mice (Figure 4C and Figure 5A). Thus, it is reasonable to deduce that Chrono interacted with Bmal1 and repressed Bmal1 transcription activity that targeted genes related to glucose metabolism.

There were some limitations to this study. Since we focused on the changes in the glycogen contents, glucose intake, and glycogen utilization in the gastrocnemius (a red/white mixed muscle fiber type) under a sedentary condition, we did not assess these variables after exercise, and did not study these variables in type I and type II muscle fibers separately. More metabolism-related data, such as the respiratory exchange ratio and mitochondrial metabolism, will be collected in a future experiment. These measurements would be desirable and would add more comprehensive insights to the scope of the study.

In conclusion, the overexpression of Chrono in skeletal muscle dramatically diminished glucose tolerance, aerobic exercise capacity, and the expression of genes involved in glucose transport, glucose oxidation, and glycogen utilization. Taken together, the results implied that Chrono played an essential role in glucose metabolism and endurance exercise capacity by binding to Bmal1.

## Figures and Tables

**Table 1:** Primer sequences.

Gene Name	Forward	Reverse
*Gys1*	5′-GAACGCAGTGCTTTTCGAGG-3′	5′-CCAGATAGTAGTTGTCACCCCAT-3′

## Data Availability

Data can be obtained from the corresponding author upon request.

## Supplementary Materials

The following supporting information can be downloaded at: https://www.mdpi.com/article/10.3390/life12081233/s1, The protocol PCR of Chrono TG mice was provided by Cyagen Biosciences Inc.
