# Supplementary material for: Skeletal-Muscle-Specific Overexpression of Chrono Leads to Disruption of Glucose Metabolism and Exercise Capacity
Source: Life (Basel). 2022 Aug 15;12(8):1233. doi: 10.3390/life12081233 (PMC9410257; doi:10.3390/life12081233)

cyagen
We help you discover life

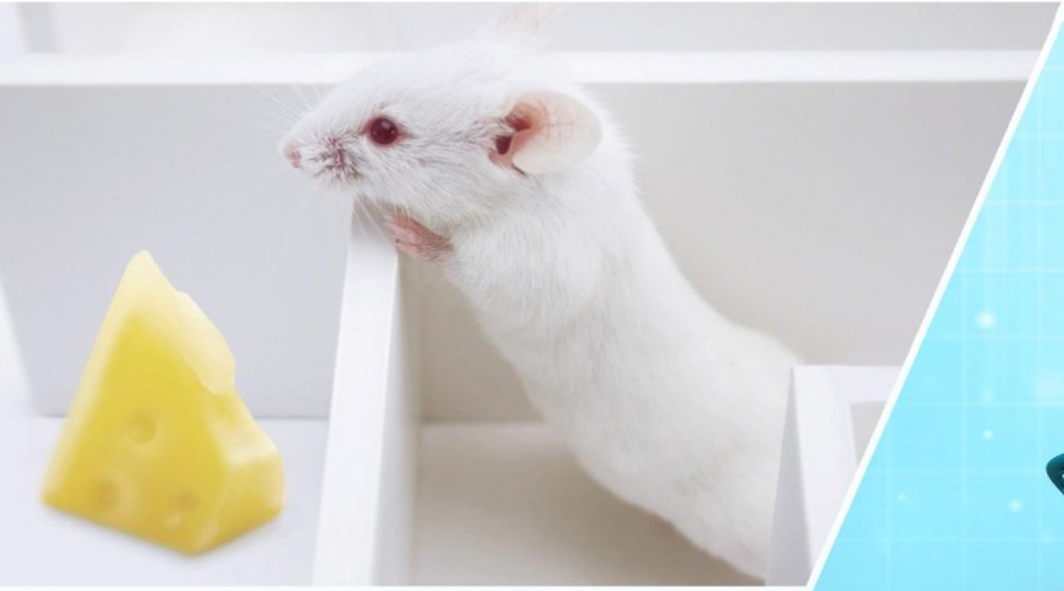

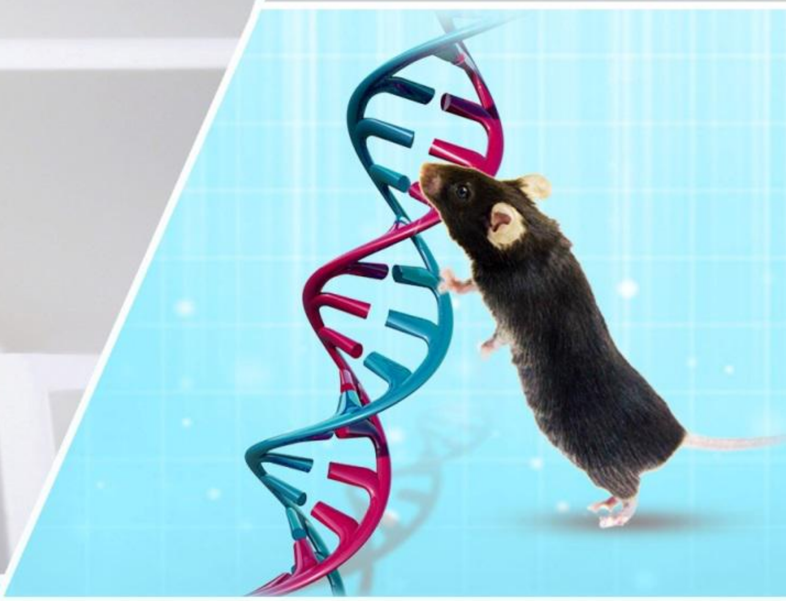

Animal Report

Quote: KICMS180530ZQ3+KICMS181002ZQ3+KICMS190412ZQ3-B
Project: Mouse Ciart-H11 Conditional Knockin

- Confidential -

Cyagen Biosciences Inc.
www.cyagen.com

S1. Method

The gRNA to Hipp11 locus, the donor vector containing “CAG promoter-loxP-Stop-loxP-kozak-mouse Ciart CDS-polyA” cassette, and Cas9 mRNA were co-injected into fertilized mouse eggs to generate targeted conditional knockin offspring. F0 founder animals were identified by PCR followed by sequence analysis, which were bred to wildtype mice to test germline transmission and F1 animal generation. Breed F1 targeted mouse with a tissue-specific Cre delete mouse to generate F2 mice that are heterozygous for a targeted allele and a hemizygous/heterozygous for the Cre transgene. Inter-cross F2 mice to generate F3 mice that are homozygous for a targeted allele and a hemizygous/heterozygous for the Cre transgene.

S2. Diagram: Breeding Scheme

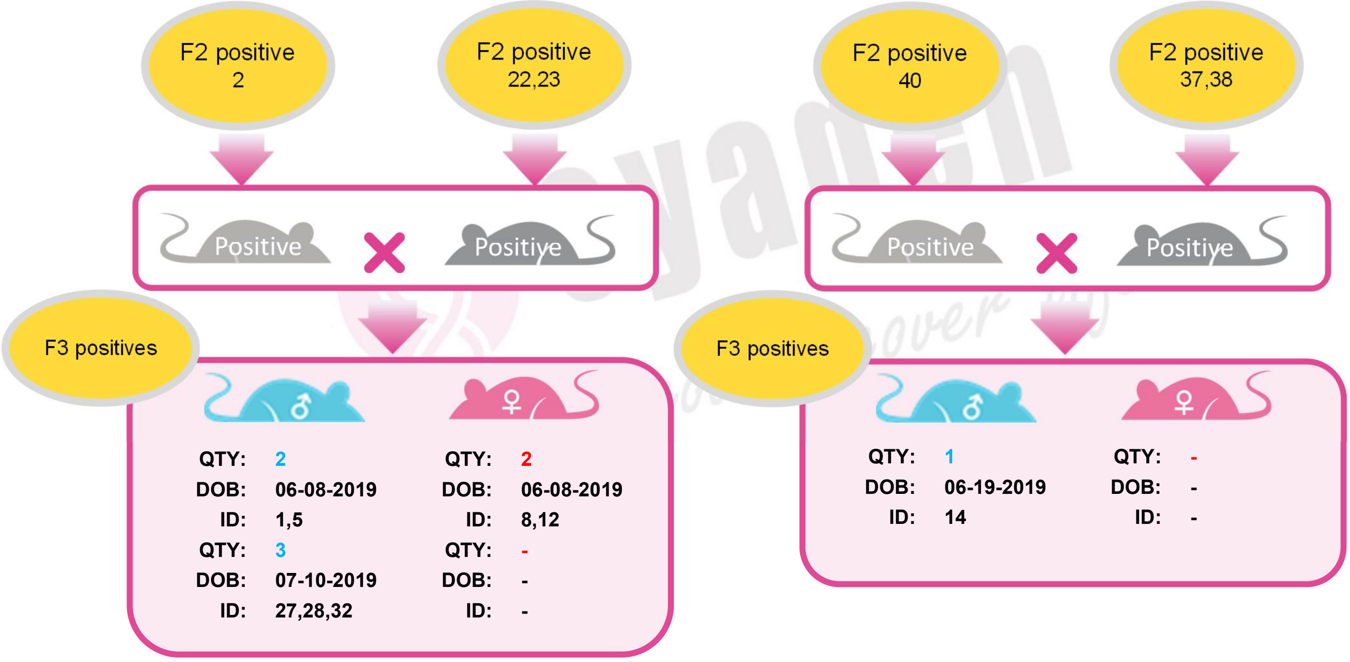

S3. Genotyping Strategy

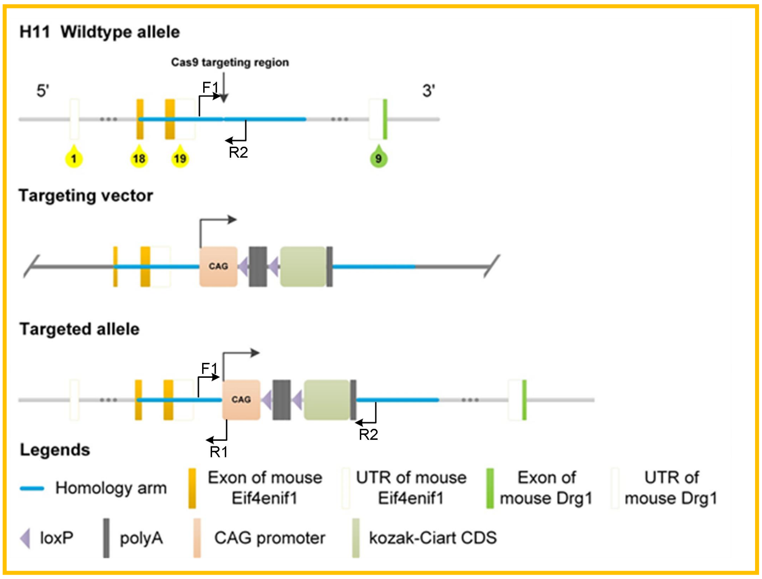

S4. PCR Screening

PCR Primers (Annealing Temperature 60.0 ºC:

Forward primer (F1: 5’-CTCTACTGGAGGAGGACAAACTG-3’

Reverse primer (R1: 5’-AGATGTACTGCCAAGTAGGAAAGTC-3’

PCR Results:

F3 animals 1, 5, 8, 12, 14, 27, 28 and 32 were identified positive by PCR screening.

Marker **PCR analysis(MT: 548 bp)**

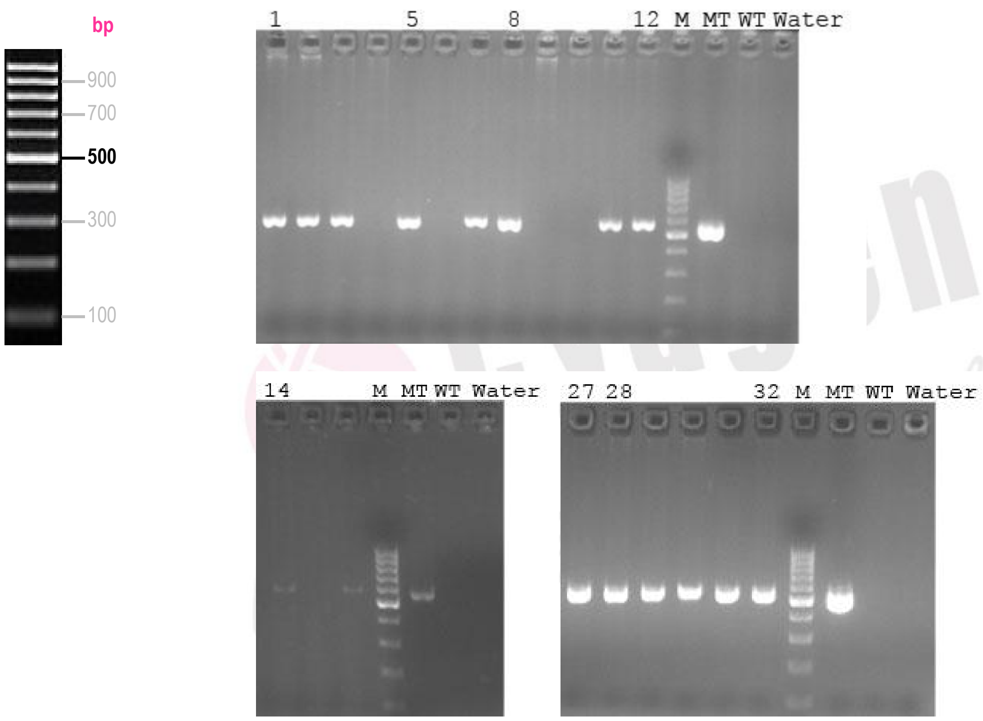

PCR Primers (Annealing Temperature 60.0 ºC):

Forward primer (F1): 5’-CTCTACTGGAGGAGGACAAACTG-3’

Reverse primer (R2): 5’-GTCTTCCACCTTTCTTCAGTTAGC-3’

PCR Results:

F3 animals 1, 5, 8, 12, 14, 27, 28 and 32 were identified by PCR screening.

Marker **PCR analysis(WT: 519 bp)**

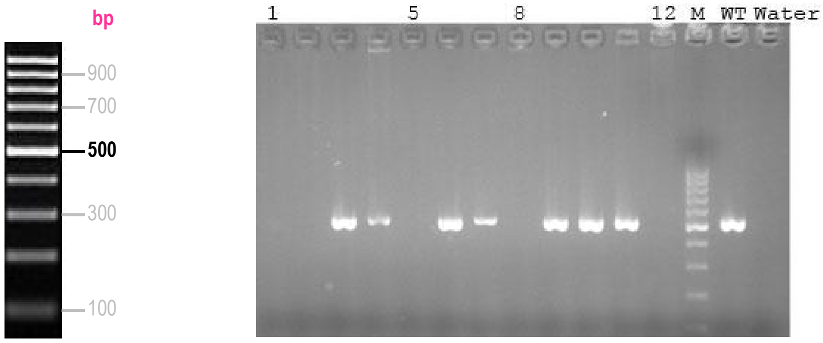

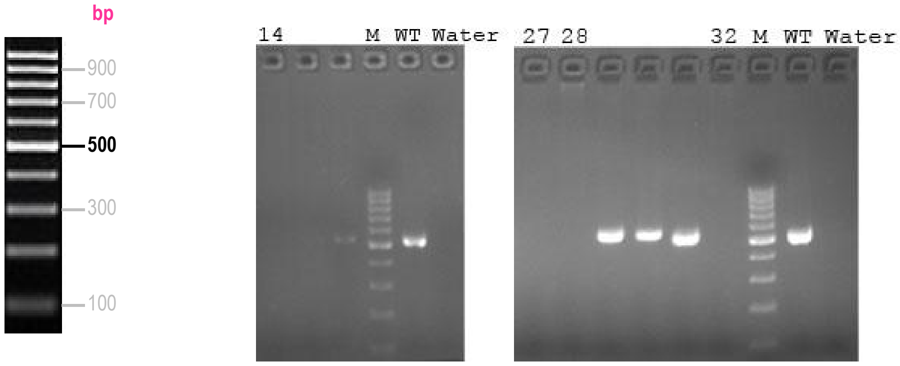

Homozygotes: 548 bp
Heterozygotes: 548 bp/519 bp
Wildtype allele: 519 bp

PCR for Myf5-Cre transgene:

F3: 5’-AACCAGAGACTCCCCAAGGT-3’
R3: 5’-ACGAAGTTATTAGGTCCCTCGAC-3’
R4: 5’-CGGCTCTTAAAGCAATGGTC-3’

Homozygotes: 120 bp
Heterozygotes: 120 bp/240 bp
Wildtype allele: 240 bp

PCR Results:

F3 animals 1, 5, 8, 12, 14, 27, 28 and 32 were identified by PCR screening.

Marker **PCR analysis(MT: 120 bp; WT: 240 bp)**

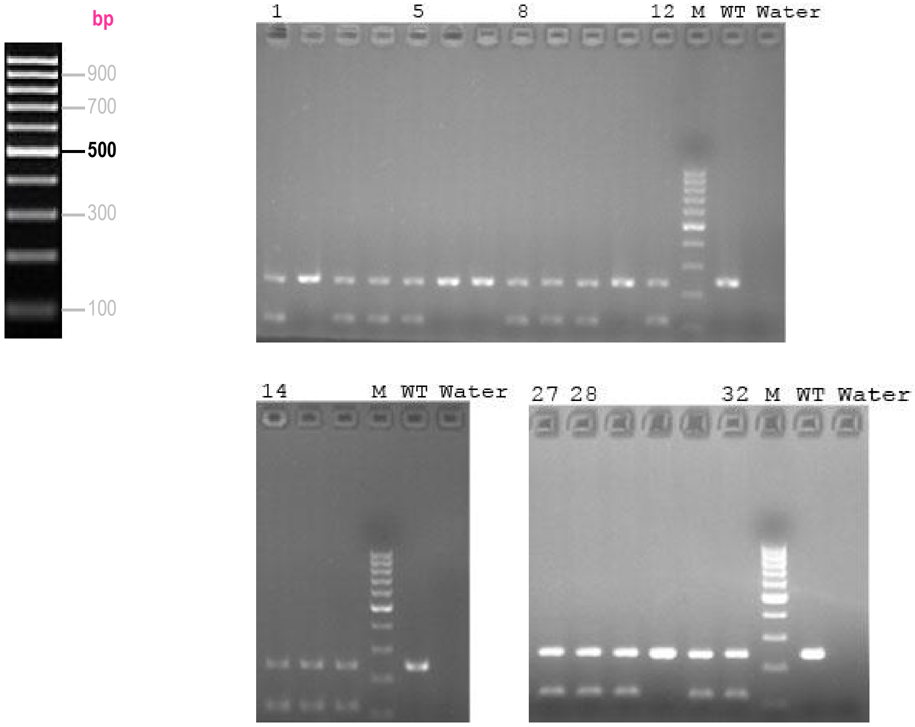

www.cyagen.com
Tel: 800-921-8930; Fax: 408-969-0336
2255 Martin Ave., Suite E. Santa Clara, CA 95050

Final Results: F3 animals 1, 5, 8, 12, 14, 27, 28 and 32 were identified homozygotes for a targeted allele and a heterozygous for the Myf5-Cre transgene.

Note:

1) PCR was carried out in 25 μL volume for 35 cycles under standard conditions, with two or three primers listed above added to each reaction.
2) Taq DNA polymerase used was P112-01.
3) Two controls used in PCR genotyping are:

- Water control: No DNA template added.
- Wildtype control: 400 ng of mouse genomic DNA.

cyagen We help you discover life

www.cyagen.com
Tel: 800-921-8930; Fax: 408-969-0336
2255 Martin Ave., Suite E. Santa Clara, CA 95050

S5. PCR Conditions Attachment

S5.1 DNA Extraction

- Method One:

We recommend that using TaKaRa MiniBEST Universal Genomic DNA Extraction kit (Ver.5.0_Code No. 9765) to gain high purity of genomic DNA.

a. Add 180 μL of Buffer GL, 20 μL of Proteinase K and 10 μL of RNase A per tail piece (2-5 mm) in a microcentrifuge tube. Be careful not to cut too much tail.

b. Incubate the tube at 56℃ overnight.

c. Spin in microcentrifuge at 12,000 rpm for 2 minutes to remove impurities.

d. Add 200 μL Buffer GB and 200 μL absolute ethyl alcohol with sufficient mixing.

e. Place the spin Column in a collection tube. Apply the sample to the spin and centrifuge at 12,000 rpm for 2 min. Discard flow-through.

f. Add 500 μL Buffer WA to the spin column and centrifuge at 12,000 rpm for 1 min. Discard flow-through.

g. Add 700 μL Buffer WB to the spin column and centrifuge at 12,000 rpm for 1 min. Discard flow-through. (Note: Make sure the Buffer WB has been premixed with 100% ethanol. When adding Buffer WB, add to the tube wall to wash off the residual salt.)

h. Repeat step g.

i. Place the spin Column in a collection tube and centrifuge at 12,000 rpm for 2 min.

j. Place the spin Column in a new 1.5ml tube. Add 50~200 μL sterilized water or elution buffer to the center of the column membrane and let the column stand 5min. (Note: Heating sterilized water or elution buffer up to 65℃ can increase the yield of elution.)

k. To elute DNA, centrifuge the column at 12,000 rpm for 2 min. To increase the yield of DNA, add the flow-through and/or 50~200 μL sterilized water or elution buffer to the center of the spin column membrane and let the column stand 5 min. Centrifuge at 12,000 rpm for 2 min.

l. Quantify to genomic DNA. Eluted genomic DNA can be quantified by electrophoresis or electrophoresis.

- Method Two:

A low-cost and sample method to gain rough genomic DNA.

a. Add 100 μL of tail digestion buffer per tail piece (2-5 mm) in a microcentrifuge tube. Be careful not to cut too much tail.

b. Incubate the tube at 56℃ overnight.

c. Incubate the tube at 98℃ for 13 minutes to denature the Proteinase K.

d. Spin in microcentrifuge at top speed for 15 minutes. Use an aliquot of supernatant straight from the tube (2 μL in a 50 μL reaction) for PCR.

Final concentration of tail digestion buffer:

- 50 mM KCl
- 10 mM Tris-HCl (pH 9.0)

www.cyagen.com
Tel: 800-921-8930; Fax: 408-969-0336
2255 Martin Ave., Suite E. Santa Clara, CA 95050

- 0.1 % Triton X-100
- 0.4 mg/mL Proteinase K

S5.2 PCR Mixture (primer concentration: 10μM):

Component	x1
ddH_2O	9.0 μl
Product primer F	1.0 μl
Product primer R	1.0 μl
Premix Taq	12.5 μl
DNA	1.5 μl
Total	25 μl

S5.3 PCR Reaction Conditions:

Step	Temp.	Time	Cycles
Initial denaturation	94 °C	3 min	
Denaturation	94 °C	30 s	35 x
Annealing	60 °C	35 s	
Extension	72 °C	35 s	
Additional extension	72 °C	5 min	

S5.4 Relevant Reagents:

**Trizma Hydrochloride Solution**	Sigma, Cat. No. T2663
**Proteinase K**	Merck, Cat. No. MK539480
**Triton X-100**	Sigma, T8787-50 mL
**2 × Taq Master Mix (Dye Plus)**	Vazyme, P112-01
**Agarose**	BIOWEST AGAROSE, REGULAR
**DNA Marker**	Thermo Scientific GeneRuler 100 bp DNA Ladder #SM0242
**0.5×TBE**	Tris Bio Basic Inc, TBO194-500g
	EDTA Shanghai Sangon, 0105-500g
	Boric Acid, Shanghai Sangon, 0588-500g

Contact Us

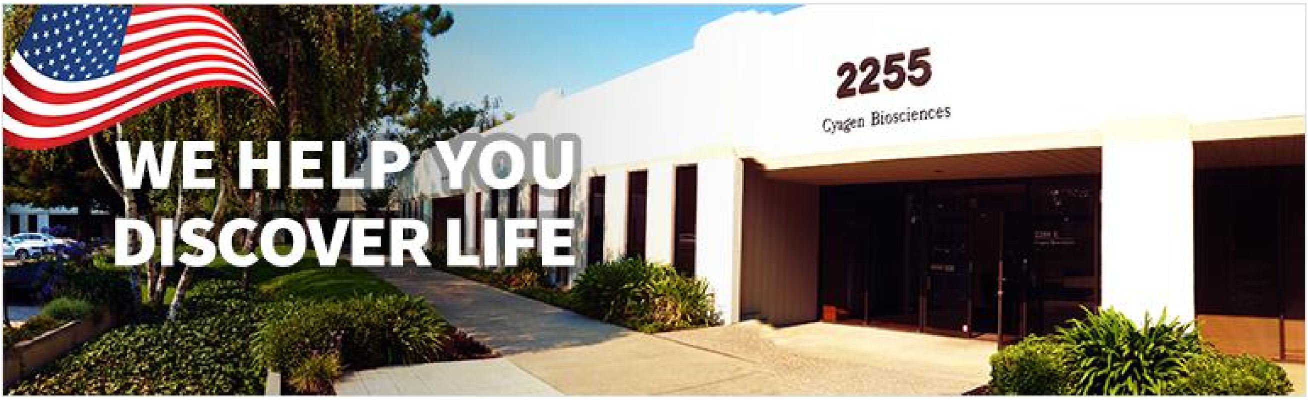

Thank you for choosing Cyagen US Inc as your research support. We sincerely hope that the mouse line generated will fulfill your expectation and will enable you to anticipate the next objective.

We also invite you to learn more about the positive impact that helping you discover life by visiting our website: www.cyagen.com.

- **North America and Europe (Headquarters)**

Cyagen US Inc.
2255 MARTIN AVE STE E
SANTA CLARA, CA 95050 – 2709, US.
Tel: US: 800-921-8930 (8-6pm PST)
+1 408-969-0306 (Int'l)
Europe: 800-793-45
+32-28085797 (Int'l)
Fax: 408-969-0336
Email: service@cyagen.com

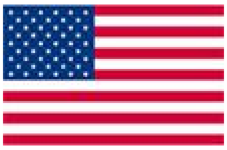

- **Asia**

Cyagen Biosciences (Guangzhou) Inc.
Building D, 3rd Floor, 3 Juquan Road, Science City
Guangzhou, 510663, China
Tel: 400-680-8038 or 86 20-28069059
Fax: +86-20-32290580
Email: info@cyagen.com

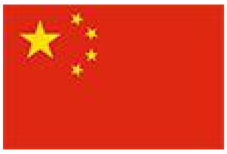

Cyagen Biosciences (Japan) Inc.
Room 4B, 1-20-10, Sugamo
Toshima ku, Tokyo 170-0002, Japan
Tel: 03-6304-1096
Fax: 03-6304-1098
Email: service@cyagen.jp

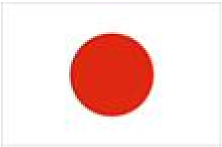

Supplement: Supplementary file 1 [file life-12-01233-s001.zip › life-1849684-supplementary.pdf]
